# Prevalence of Neonatal Sepsis in Ethiopia: A Systematic Review and Meta-Analysis

**DOI:** 10.1155/2020/6468492

**Published:** 2020-04-15

**Authors:** Moges Agazhe Assemie, Muluneh Alene, Lieltwork Yismaw, Daniel Bekele Ketema, Yonas Lamore, Pammla Petrucka, Simegn Alemu

**Affiliations:** ^1^Department of Public Health, College of Health Sciences, Debre Markos University, Debre Markos, Ethiopia; ^2^Department of Environmental Health, College of Health Sciences, Debre Markos University, Debre Markos, Ethiopia; ^3^College of Nursing, University of Saskatchewan, Saskatoon, Canada; ^4^School of Life Sciences and Bioengineering, Nelson Mandela African Institute of Science and Technology, Arusha, Tanzania

## Abstract

**Introduction:**

Neonatal sepsis is a systemic infection occurring in infants during the first 4 weeks of life and is a major cause of mortality and morbidities of newborns due to their age-related weak and immature immune systems. In Ethiopia, despite many studies being conducted on neonatal sepsis, the reported findings are inconsistent. The aim of this study is to determine the prevalence of neonatal sepsis to enhance the utility and interpretation of the evidence.

**Methods:**

An extensive systematic review and meta-analysis were performed to extract studies on the prevalence of neonatal sepsis in Ethiopia. The PubMed, Cochrane Library, ScienceDirect, Web of Science, and Google Scholar were systematically searched. Two independent authors selected and extracted the data from each included article. The heterogeneity of included studies was assessed using the Higgins *I*^2^ test, and a random-effects model was performed in Stata/se Version 14.

**Results:**

Eighteen studies with a sample size of 10,495 study subjects were included with a reported range of neonatal sepsis from 17% to 78%. The pooled prevalence of neonatal sepsis was 45% (95% CI: 35, 55; *I*^2^ = 99.3%, *p* < 0.01). Early onset neonatal sepsis was found to have a prevalence of 75.4% (95% CI: 68.3, 82.6). Subgroup analysis in the study area (i.e., by region) was calculated revealing the highest neonatal sepsis in Amhara region at 64.4% (95% CI: 44.9, 84.0) and the lowest in Southern Nations, Nationality, and People at 28% (95% CI: 16, 40).

**Conclusion:**

In this review, the prevalence of neonatal sepsis in Ethiopia was found to be high, especially in terms of early onset neonatal sepsis. As a result of the findings, it is important to consider the early and optimal points for interventions to better manage the prevalence and outcomes of neonatal sepsis. Further research is needed to investigate the neonatal sepsis status at different regions and associated factors for neonatal sepsis not yet studied.

## 1. Introduction

The neonatal period is the most vulnerable time for children's survival. Globally every year about 4 million children die in the first 4 weeks of life, of which 99% of the deaths occur in low- and middle-income countries and of which 75% are considered avoidable [1]. Even though neonatal mortality shows a declining trend over the last 20 years from 50.6 per 1000 live births in 1998 to 28.9 per 1000 live births in 2017 [2], Ethiopia continuous to struggle with a prevalence of about 42% or 81,000 newborn deaths every year [1]. Thus, neonatal sepsis is a major cause of neonatal mortality due to a higher risk of infection because of their weak and immature immune systems related to their age [3, 4].

Neonatal sepsis is a systemic infection occurring in infants at the first 4 weeks of life which can be classified as either early or late onset sepsis [1, 5]. Clinical signs and symptoms of sepsis in newborns vary by gestational age and severity of infection. It is more common for a septic infant to be hypothermic upon presentation [6]. Among all major causes of neonatal deaths, sepsis accounts for 25% of all neonatal deaths in sub-Saharan Africa and southern Asia [7]. Even though there are some improvements to access essential preventive, primary child health care services and sector training [1], neonatal sepsis is still the major cause of newborn deaths resulting in more than one-third of all neonatal deaths [1, 8].

To date, there are inconsistent findings and no systematic review and meta-analysis have been done to enhance the quality and consistency of the evidence. Therefore, the aim of this study was to consider the evidence to determine the prevalence of neonatal sepsis in Ethiopia while serving as a baseline for clinicians and policy makers to design future infrastructure and system strengthening to improve the quality of health.

## 2. Methods

### 2.1. Search Strategy

To locate potential articles, PubMed, Google Scholar, Cochrane Library, Web of Science, and ScienceDirect were comprehensively searched between January 27 and June 3, 2019. We extended our search by reviewing reference lists of eligible articles, hand searching for grey literature, and other important literature collections including the Addis Ababa Digital Library and Saint Mary's University repositories. The search protocol was formulated by using common keywords: prevalence AND associated factors AND pediatrics OR infant OR newborn OR neonate's sepsis AND Ethiopia (MeSH Terms). The data selection of this systematic review was presented according to the preferred reporting items for systematic reviews and meta-analysis (PRISMA) guidelines [9]. However, it was not registered on the prospective registration of systematic review and meta-analysis (PROSPERO), which is addressed in Limitations of the Study.

### 2.2. Eligibility

Included articles were both published and unpublished full text observational study designs reporting the prevalence of neonatal sepsis in Ethiopia, whereas case reports, national reports, clinical studies, and reviews were excluded.

### 2.3. Outcome Variables

Neonatal sepsis is the main outcome of the study and calculated as the total number of sepsis cases divided by the total number of live birth infants in the study multiplied by 100. Neonatal sepsis can be classified as early onset sepsis acquired from birth to 7 days and late onset sepsis acquired after delivery in the normal newborn nursery, the neonatal intensive care unit, or the community (8 to 30 days). Thus, meta-analysis was performed for two or more studies reporting the same outcomes [10].

### 2.4. Data Selection and Extraction

Two independent reviewers (SA and YL) screened the downloaded articles and extracted all necessary data from included articles; discussions and mutual consensus were used when discrepancies arose. The extraction format included primary author, study design, classification of sepsis onset, data source, health facility, study area/region, study period, publication year, quality score, sample size, and prevalence with 95% confidence interval.

### 2.5. Quality Assessment and Appraisal

We had performed a critical appraisal of the research evidence to assess the methodological quality of a study to determine the extent to which a study has addressed the possibility of bias in its design, conduct, and analysis using a standardized data appraisal format adapted from the Joanna Briggs Institute (JBI) checklist [11].

Moreover, to assess the quality of each primary study, the Newcastle-Ottawa Scale (NOS) for cross-sectional studies was adopted [12]. The tool has three main components and uses a star grading system. The first component has possible five stars and considers the credibility on the selection of study groups. The second section of the tool deals with the comparability of the groups with a possibility of two stars. The third section of the grading system focuses on the ascertainment for either exposure or outcome of each original study with a possibility of three stars to be assessed. In addition, quality appraisal of included studies was evaluated by two authors (MA and LY) independently and any discrepancy was resolved by a third author (MAA). Articles with a NOS score of ≥5 stars out of 10 were considered as high quality [13] for the purposes of our work (Supplementary Table).

### 2.6. Risk of Bias Assessment

The risk of bias assessment of included articles was evaluated by two authors (LY and SA) independently using the Hoy et al. (2012) adapted tool for prevalence studies which consists of 10 items addressing four domains of bias plus a summary risk of bias assessment [14]. Any discrepancy was resolved by discussion and mutual consensus mediated by a third author (MAA).

### 2.7. Data Processing and Analysis

Data were extracted in a Microsoft™ Excel spreadsheet, and analysis was carried out using Stata/se Version 14 statistical software. Heterogeneity among reported prevalence was assessed by computing *p* values of Higgins's *I*^2^ statistics; *I*^2^ was considered as significant at a *p* value < 0.10 [15]. The DerSimonian and Laird's random-effects meta-analysis model was used to determine the pooled effect size, since the true effect is not the same in all studies [16].

We deal heterogeneity with subgroup analysis, metaregression, and sensitivity analysis. Subgroup analysis was done based on study settings. In addition, an effort to understand the sources of heterogeneity, univariate metaregression analysis was conducted for sample size, publication year, study design, quality score, and midyear study period. Metaregression was used instead of subgroup analyses since it allowed for the use of continuous covariates and permitted the inclusion of more than one covariate at a time. Moreover, sensitivity analysis was computed to assess the influence of a single study on the pooled estimates.

A forest plot was used to describe pooled prevalence with 95% confidence intervals. The size of each box indicated the weight of the study, while each crossed line refers to a 95% confidence interval with the mean effect at the center. The possibility of publication bias was assessed visually with funnel plots, and the objectivity test of Egger's test with *p* value less than 0.05 was considered evidence of publication bias.

## 3. Results

### 3.1. Study Selection

**Figure 1:**
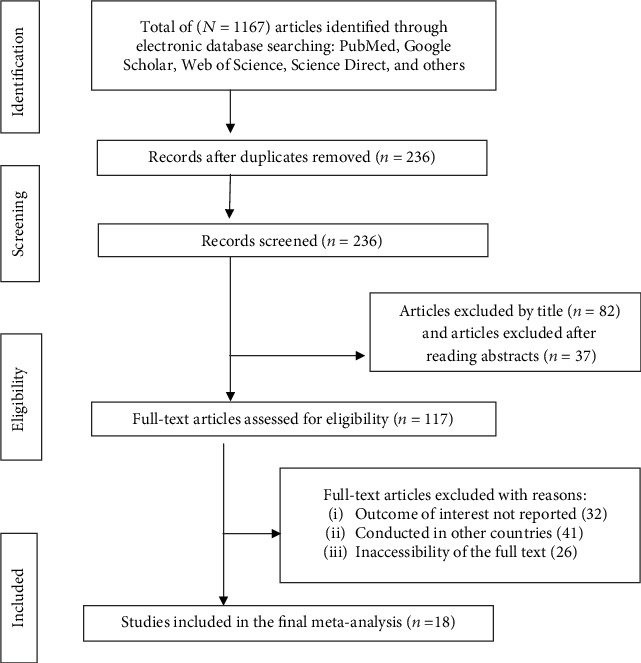
PRISMA study selection flow diagram for systematic review and meta-analysis on neonatal sepsis in Ethiopia, 2019 (*n* = 18).

We followed the PRISMA guideline to present the findings of this review. We extracted 1167 articles regarding neonatal sepsis using PubMed, Google Scholar, Cochrane Library, Web of Science, and ScienceDirect. After removing duplicates, 236 articles were screened of which 119 were excluded after reading the title and abstracts. The remaining 117 full text articles were assessed for eligibility. Eighteen studies met the eligibility criteria and were included in the final analysis, as shown in the chart of study selection process (Figure 1).

### 3.2. Descriptive Summary of Included Neonatal Sepsis Articles

As described in Table 1, these 18 studies were cross-sectional and cohort study designs published between March 2005 and March 18, 2019, along with selected grey literature. In the current systematic review and meta-analysis, 10,495 neonates were included. The sample size by study ranged from 169 in Tikur Anbesa Specialized Hospital (TASH), Addis Ababa [17], to 3418 in Dilchora Referral Hospital (DRH), Dire Dawa City Administration [18]. The prevalence of neonatal sepsis reported was between 22% in Karamara General Hospital (KGH), Somalia region [19], and 78% in Shashemene Referral Hospital (SRH), Oromia region [20].

### 3.3. Prevalence of Neonatal Sepsis in Ethiopia

**Figure 2:**
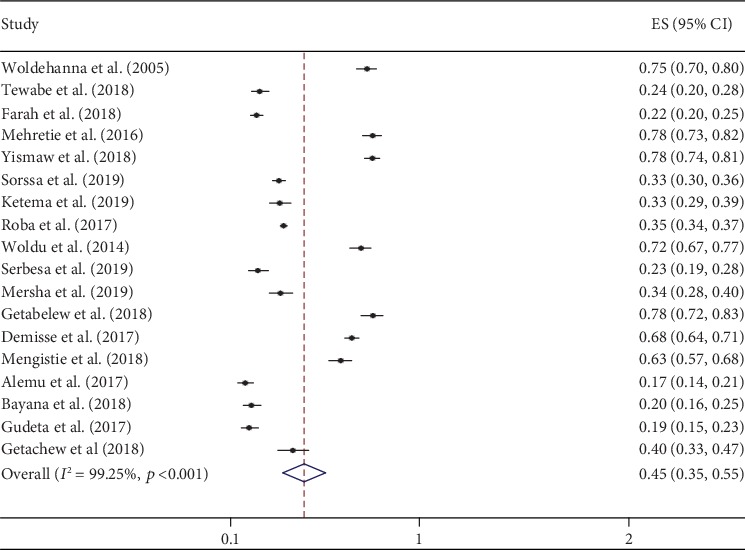
Forest plot of the pooled prevalence of neonatal sepsis in Ethiopia, 2019 (*n* = 18).

**Figure 3:**
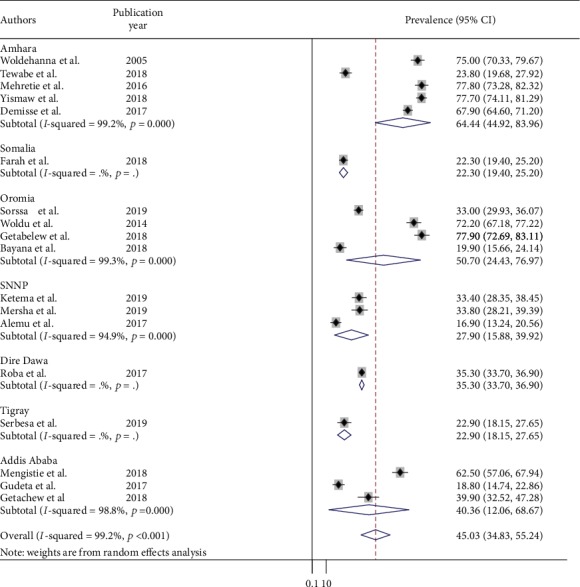
Subgroup analysis of neonatal sepsis by study area (region) of Ethiopia, 2019 (*n* = 18).

Prevalence of neonatal sepsis was found to be 45% (95% CI: 35, 55; *I*^2^ = 99.3%, *p* < 0.01) which indicates high heterogeneity (Figure 2). Hence, subgroup analysis, based on the study regions, was computed and did not show a significant level of difference/heterogeneity for neonatal sepsis. The highest prevalence was found in Amhara region to be 64% (95% CI: 45, 84), followed by the Oromia region at 51% (95% CI: 24, 77), whereas the lowest prevalence was found in Southern Nations, Nationality, and People at 28% (95 CI: 16, 40) (Figure 3).

**Figure 4:**
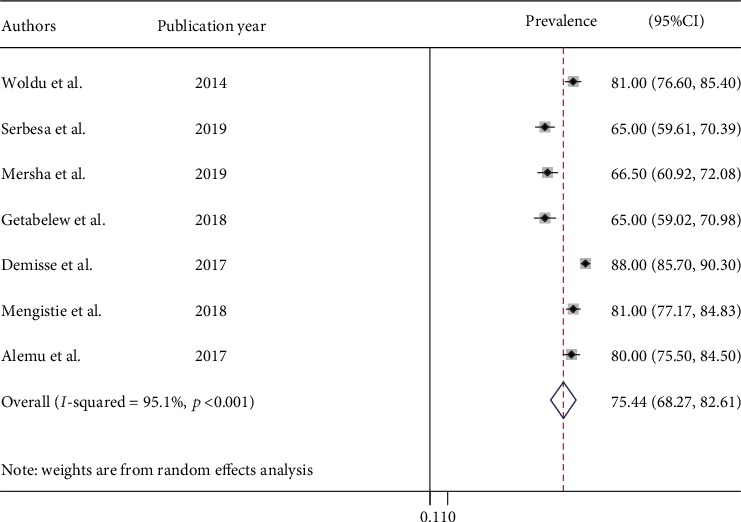
Prevalence of early onset of neonatal sepsis in Ethiopia, 2019 (*n* = 7).

Among the included studies, seven of the studies reported neonatal sepsis as early and late onset neonatal sepsis classification. Thus, early onset neonatal sepsis was reported in the range of 65% SRH, Oromia, and 88% in Gondar Teaching Hospital (GTH), Amhara. Thus, we found 75.4% (95% CI: 68.3, 82.6) pooled early onset neonatal sepsis (Figure 4).

In addition, univariate metaregression analysis was conducted to identify possible sources of heterogeneity for midyear study period, publication year, quality score, and sample size. Again, all of these covariates were found to be statistically nonsignificant (Table 2).

**Figure 5:**
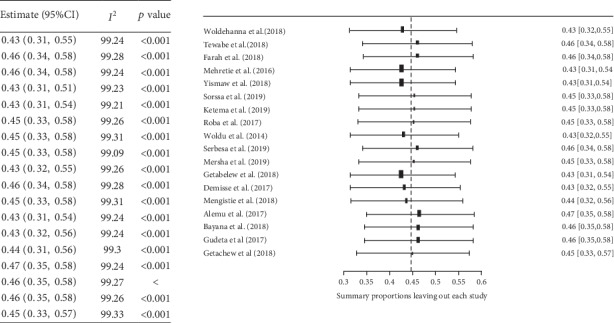
Sensitivity analysis for neonatal sepsis eligible studies in Ethiopia, 2019 (*n* = 18).

Moreover, sensitivity analysis was computed to evaluate whether the exclusion of any single study altered the magnitude or statistical results of the summary estimate. None of the studies influenced the summary pooled estimates (Figure 5).

**Figure 6:**
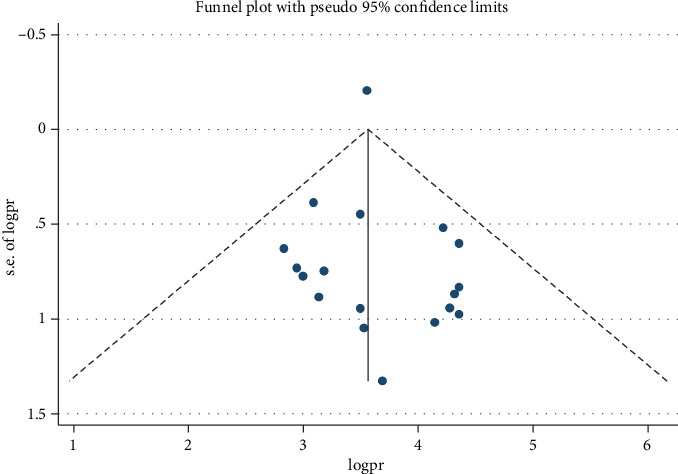
Funnel plot with 95% confidence limits of the pooled prevalence of neonatal sepsis in Ethiopia, 2019 (*n* = 18).

Publication bias was assessed by a funnel plot and absence of bias was represented by substantial symmetry (Figure 6). To confirm the absence of publication bias, Egger's test was employed and did not show the presence of bias (*p* = 0.627).

## 4. Discussion

Neonates are at a higher risk of infection because of their developmentally weak and immature immune systems. In this study, the pooled prevalence of neonatal sepsis for Ethiopia was found to be 45%, while early onset neonatal sepsis acquired before or during delivery accounted for 75.4%.

This study revealed that pooled prevalence of neonatal sepsis in Ethiopia is consistent with the findings from Cameroon (37.9%) [35], Tanzania (38.9%) [36], and Egypt (45%) [37]. However, our finding is higher than previous studies reported in Iran (18.4%) [38], Mexico (4.3%) [39], and Egypt (8.6%) [40]. This variation could be due to unique cultural features of the population, local obstetrics and neonatal practices, socioeconomic and sexual practice, hygiene, and nutritional differences over settings [41] as well as due to clinical features for sepsis identification, study methodology, and sample size difference which we observed during our study.

The subgroup analysis of neonatal sepsis studies based on region of the country found 64% (95% CI: 45, 84) in Amhara region where studies were from specialized hospital chart review followed by the Oromia region at 51% (95% CI: 24, 77), whereas the lowest was observed in Southern Nations, Nationality, and People at 28%(95% CI: 16, 40) primary study at district hospital. The possible explanation for this variation could be due to the differences in health facility, study design, and sample size variation across studies.

### 4.1. Limitations of the Study

A number of the studies included in this review had a relatively small sample size which may decrease the power of the study. Secondly, there are studies only from five regions and two city administrations of Ethiopia, which restrict the representativeness of the study. This study also emphasized only the prevalence of neonatal sepsis. In addition, this study is not registered in PROSPERO which could compromise transparency and credibility of the study.

## 5. Conclusion

In this review, the prevalence of neonatal sepsis in Ethiopia was found to be high, especially in terms of early onset neonatal sepsis. As a result of the findings, it is important to consider the timing and optimal points for interventions to better manage the occurrence of neonatal sepsis. Further research is needed to investigate the neonatal sepsis status at different regions not yet studied and associated factors for neonatal sepsis.

## Figures and Tables

**Table 1:** Descriptive summary of studies included in the systematic review and meta-analysis of neonatal sepsis in Ethiopia.

Authorship (reference)	Study period	Publication (yr)	Study design	Facility name	Study area (region)	Source of data	Sample	Early onset sepsis	Late onset sepsis	Prevalence (95% CI)
Woldehanna and Idejene [21]	1/9/1994–31/8/1999	2005	Retrospective	GTH	Gondar (Amhara)	Chart review	330	NA	NA	75 (70, 80)
Tewabe et al. [22]	30/4/16-30/5/2016	2018	Retrospective	FHRH	Bahir Dar (Amhara)	Chart review	410	NA	NA	24 (20, 28)
Farah et al. [19]	8/2014-2017	2018	Retrospective	KGH	Karamara (Somalia)	Char review	792	NA	NA	22 (19, 25)
Kokeb and Desta [23]	1/1/2014- 31/3/2014	2016	Cross-sectional	GTH	Gondar (Amhara)	Primary	325	NA	NA	78 (73, 82)
Yismaw and Tarekegn [24]	1/2016–3/2018	2018	Retrospective	GTH	Gondar (Amhara)	Chart review	516	NA	NA	78 (74, 81)
Sorsa et al. [25]	4/2016–5/2017	2019	Cross-sectional	ATH	Asela (Oromia)	Primary	902	NA	NA	33 (30, 36)
Ketema et al. [26]	9-10,2017	2019	Retrospective	JGH	Jinka (SNNP)	Chart review	335	NA	NA	33 (28,39)
Roba and Diro [18]	1/1/2013-10/1/2017	2017	Retrospective	DRH	Dire Dawa	Chart review	3418	NA	NA	35 (34, 37)
Woldu et al. [27]	15/4/2014-15/10/2014	2014	Cross-sectional	BGH	Bishoftu (Oromia)	Primary	306	81	19	72 (67, 77)
Serbesa and Iffa [28]	2–5,2017	2019	Cross-sectional	ARH	Mekelle (Tigray)	Primary	301	65.1	34.9	23 (18, 28)
Mersha et al. [29]	22/4/2018-29/6/2018	2019	Cross-sectional	WSTH & SCH	Wolaita (SNNP)	Primary	275	66.5	34.5	34 (28, 39)
Getabelew et al. [20]	2/2016-2/2017	2018	Cross-sectional	SRH and MGH	Shashemene (Oromia)	Primary	244	65	35	78 (73, 83)
Demisse et al. [30]	1/12/2015–31/8/2016	2017	Retrospective	GTH	Gondar (Amhara)	Chart review	769	87.7	12.3	68 (65, 71)
Mengistie et al. [31]	1-2/2018	2018	Cross-sectional	HTH & AGH	Hawassa (SNNP)	Primary	402	80.9	19.1	62 (57, 68)
Alemu [32]	9/2016 2/2017	2017	Retrospective	TASH	Addis Ababa	Chart review	304	80	20	17 (13, 21)
Bayana et al. [33]	1/1/2016-31/12/2017	2018	Retrospective	JTH	Jimma (Oromia)	Chart review	341	NA	NA	20 (16, 25)
Gudeta [34]	11/8/2014-1/11/2016	2017	Retrospective	TASH	Addis Ababa	Chart review	356	NA	NA	19 (15, 23)
Getachew [17]	11/2017-7/2018	2018	Cross-sectional	TASH	Addis Ababa	Primary	169	NA	NA	40 (32, 47)

MGH = Melkaoda General Hospital; WSTH = Wolaita Sodo Teaching Hospital; SCH = Sodo Christian Hospital; HTH = Hawassa Teaching Hospital; AGR = Adare General Hospital; ARH = Ayder Referral Hospital; BGH = Bishoftu General Hospital; FHRH = Felege Hiwot Referral Hospital; ATH = Asela Teaching Hospital; JGH = Jinka General Hospital; JTH = Jimma Teaching Hospital.

**Table 2:** Univariate metaregression for related factors to heterogeneity of neonatal sepsis.

Variables	Coefficient	*p* value
Sample size	-0.0000	0.62
Publication year	-0.0342	0.053
Quality score	-0.0055	0.97
Midyear study period	-0.0177	0.15

## Data Availability

Minimal data can be accessed upon request from first author.

## Supplementary Materials

Supplementary Materials Table S2: assessing the risk of bias for the included studies.

## References

[1] Federal Ministry of Health of Ethiopia (2014). *Neonatal Intensive Care Unit (NICU) Training Participants’ Manual*.

[2] UNICEF *Monitoring the situation of children and women 2017*.

[3] Fleischmann-Struzek C., Goldfarb D. M., Schlattmann P., Schlapbach L. J., Reinhart K., Kissoon N. (2018). The global burden of paediatric and neonatal sepsis: a systematic review. *The Lancet Respiratory Medicine*.

[4] Wu J. H., Chen C. Y., Tsao P. N., Hsieh W. S., Chou H. C. (2009). Neonatal sepsis: a 6-year analysis in a neonatal care unit in Taiwan. *Pediatrics and Neonatology*.

[5] Krugman S., Gershon A., Hotez P. J., Katz S. L. (2004). *Krugman’s Infectious Diseases of Children*.

[6] Simonsen K. A., Anderson-Berry A. L., Delair S. F., Davies H. D. (2014). Early-onset neonatal sepsis. *Clinical Microbiology Reviews*.

[7] WHO (2017). *Preventable maternal and neonatal sepsis a critical priority for WHO and Global Sepsis Alliance*.

[8] Berhanu D., Avan B. I. (2014). *Community Based Newborn Care Baseline Survey Report Ethiopia*.

[9] Liberati A., Altman D. G., Tetzlaff J. (2009). The PRISMA statement for reporting systematic reviews and meta-analyses of studies that evaluate health care interventions: explanation and elaboration. *Journal of Clinical Epidemiology*.

[10] Feldmann M., Ullrich C., Bataillard C. (2019). Neurocognitive outcome of school-aged children with congenital heart disease who underwent cardiopulmonary bypass surgery: a systematic review protocol. *Systematic Reviews*.

[11] Munn Z., Moola S., Riitano D., Lisy K. (2014). The development of a critical appraisal tool for use in systematic reviews addressing questions of prevalence. *International Journal of Health Policy and Management*.

[12] Chikuse B., Chirwa E., Maluwa A., Malata A., Odland J. (2012). Midwives’ adherence to guidelines on the management of birth asphyxia in Malawi. *Open Journal of Nursing*.

[13] Wells G. (2004). The Newcastle-Ottawa Scale (NOS) for assessing the quality of nonrandomised studies in meta-analysis. https://ci.nii.ac.jp/naid/10020590649/.

[14] Hoy D., Brooks P., Woolf A. (2012). Assessing risk of bias in prevalence studies: modification of an existing tool and evidence of interrater agreement. *Journal of Clinical Epidemiology*.

[15] Rucker G., Schwarzer G., Carpenter J. R., Schumacher M. (2008). Undue reliance on *I*^2^ in assessing heterogeneity may mislead. *BMC Medical Research Methodology*.

[16] Borenstein M., Hedges L. V., Higgins J. P. T., Rothstein H. R. (2010). A basic introduction to fixed-effect and random-effects models for meta-analysis. *Research Synthesis Methods*.

[17] Getachew A. (2018). *Prevalence of Periventricular-Intraventricular Hemorrhage Diagnosed on Transcranial Ultrasound among Preterm Neonates Admitted to the NICU in Tikur Anbessa Specialized Hospital*.

[18] Roba A., Diro D. (2017). Morbidities, rate and time trends of neonatal mortality in Dilchora Referral Hospital, Dire Dawa, Ethiopia, 2012-2017. *Austin Medical Sciences*.

[19] Elmi Farah A., Abbas A. H., Tahir Ahmed A. (2018). Trends of admission and predictors of neonatal mortality: a hospital based retrospective cohort study in Somali region of Ethiopia. *PLoS One*.

[20] Getabelew A., Aman M., Fantaye E., Yeheyis T. (2018). Prevalence of neonatal sepsis and associated factors among neonates in neonatal intensive care unit at selected governmental hospitals in Shashemene Town, Oromia Regional State, Ethiopia, 2017. *International Journal of Pediatrics*.

[21] Woldehanna T. D., Idejene E. T. (2005). Neonatal mortality in a teaching hospital, North Western Ethiopia. *The Central African Journal of Medicine*.

[22] Tewabe T., Mehariw Y., Negatie E., Yibeltal B. (2018). Neonatal mortality in the case of Felege Hiwot referral hospital, Bahir Dar, Amhara Regional State, North West Ethiopia 2016: a one year retrospective chart review. *Italian Journal of Pediatrics*.

[23] Kokeb M., Desta T. (2016). Institution based prospective cross-sectional study on patterns of neonatal morbidity at Gondar University Hospital Neonatal Unit, North-West Ethiopia. *Ethiopian Journal of Health Sciences*.

[24] Yismaw A. E., Tarekegn A. A. (2018). Proportion and factors of death among preterm neonates admitted in University of Gondar comprehensive specialized hospital neonatal intensive care unit, Northwest Ethiopia. *BMC Research Notes*.

[25] Sorsa A., Fruh J., Stotter L., Abdissa S. (2019). Blood culture result profile and antimicrobial resistance pattern: a report from neonatal intensive care unit (NICU), Asella teaching and referral hospital, Asella, south East Ethiopia. *Antimicrobial Resistance and Infection Control*.

[26] Erkihun K., Mesfin M., Direslegn M., Sultan H., Negussie B. (2019). Determinants of neonatal sepsis among neonates admitted in a neonatal intensive care unit at Jinka General Hospital, Southern Ethiopia. *International Journal of Nursing and Midwifery*.

[27] Woldu M. A., Guta M. B., Lenjisa J. L., Tegegne G. T., Tesafye G., Dinsa H. (2017). Assessment of the incidence of neonatal sepsis, its risk factors, antimicrobials use and clinical outcomes in Bishoftu General Hospital, neonatal intensive care unit, Debrezeit-Ethiopia. *International Journal of Contemporary Pediatrics*.

[28] Serbesa M. L., Iffa M. T. (2019). Diagnose at admission and factors associated with management outcome of neonate in Ayder Referral Hospital, northern Ethiopia: institutional based cross-sectional record review study. *Journal of Pediatrics & Neonatal Care*.

[29] Mersha A., Worku T., Shibiru S. (2019). Neonatal sepsis and associated factors among newborns in hospitals of Wolaita Sodo Town, Southern Ethiopia. *Research and Reports in Neonatology*.

[30] Demisse A. G., Alemu F., Gizaw M. A., Tigabu Z. (2017). Patterns of admission and factors associated with neonatal mortality among neonates admitted to the neonatal intensive care unit of University of Gondar Hospital, Northwest Ethiopia. *Pediatric Health, Medicine and Therapeutics*.

[31] Mengistie B., Geda B., Ahmed A. (2018). *Neonatal Sepsis and Associated Factors among Neonates in Public Hospitals of Hawassa City Administration*.

[32] Alemu M. (2017). *Assessment of Pattern of Admission and Outcome of Neonates Admitted to Neonatal Intensive Care Unit at Tikur Anbessa Sepecialized Teaching Hospital, Addis Ababa, Ethiopia, 2017*.

[33] Bayana E. (2018). *Pattern of Disease, Outcome & Associated Factors Among Neonates Admitted to Neonatal Intensive Care Unit*.

[34] Gudeta H. (2017). *Assesment of Magnitude and Associated Factors of Neonatal Hyperbilirubinemia at Neonatal Intensive Care Unit of Tikur Anbessa Specialized Hospital, Addis Ababa, Ethiopia, 2017*.

[35] Mah Mungyeh E., Chiabi A., Tchokoteu Pouasse F. L. (2014). Neonatal mortality in a referral hospital in Cameroon over a seven year period: trends, associated factors and causes. *African Health Sciences*.

[36] Kayange N., Kamugisha E., Mwizamholya D. L., Jeremiah S., Mshana S. E. (2010). Predictors of positive blood culture and deaths among neonates with suspected neonatal sepsis in a tertiary hospital, Mwanza-Tanzania. *BMC Pediatrics*.

[37] Shehab el-Din E. M., el-Sokkary M. M., Bassiouny M. R., Hassan R. (2015). Epidemiology of neonatal sepsis and implicated pathogens: a study from Egypt. *BioMed Research International*.

[38] Rakhsha M., Pourali L., Ayati S., Boskabadi H., Kazemi K., Shakeri M. T. (2016). Effective maternal and neonatal factors associated with the prognosis of preterm infants. *Journal of Patient Safety & Quality Improvement*.

[39] Leal Y. A., Álvarez-Nemegyei J., Velázquez J. R. (2012). Risk factors and prognosis for neonatal sepsis in southeastern Mexico: analysis of a four-year historic cohort follow-up. *BMC Pregnancy and Childbirth*.

[40] Medhat H., Khashana A., El Kalioby M. (2017). Incidence of neonatal infection in South Sinai, Egypt. *International Journal of Infection*.

[41] Wilson C. B., Nizet V., Remington J. S., Klein J. O., Maldonado Y. (2010). *Infectious Diseases of the Fetus and Newborn E-Book*.

