# Supplementary material for: Prevalence of Neonatal Sepsis in Ethiopia: A Systematic Review and Meta-Analysis
Source: Int J Pediatr. 2020 Apr 15;2020:6468492. doi: 10.1155/2020/6468492 (PMC7180396; doi:10.1155/2020/6468492)
Supplement: Supplementary Materials — Table S2: assessing the risk of bias for the included studies. [file 6468492.f1.docx]

| S2 Table S2: Assessing the risk of bias for the included studies | | | |  |
| --- | --- | --- | --- | --- |
| **Author (publication year)** | **Selection** | **Comparability** | **Outcome** | **Quality** |
| Woldehanna et al.(2005) | *** | * | *** | High |
| Tewabe et al.(2018) | **** | * | *** | High |
| Farah et al.(2018) | **** | * | ** | High |
| Mehretie et al.(2016) | ** | * | ** | Low |
| Yismaw et al.(2018) | *** | ** | ** | High |
| Sorssa et al.(2019) | **** | * | ** | High |
| Ketema et al. (2019) | **** | * | ** | High |
| Roba et al.(2017) | ** | * | ** | Low |
| Woldu et al.(2014) | *** | ** | ** | High |
| Serbesa et al.(2019) | *** | * | *** | High |
| Mersha et al.(2019) | **** | * | ** | High |
| Getabelew et al.(2018) | *** | * | *** | High |
| Demisse et al.(2017) | **** | * | ** | High |
| Mengistie et al.(2018) | ** | * | ** | Low |
| Alemu et al.(2017) | ** | * | ** | High |
| Bayana et al.(2018) | ** | ** | ** | Low |
| Gudeta et al.(2017) | **** | * | ** | High |
| Getachew et al.(2018) | *** | ** | ** | High |
| **Risk of bias assessment tool** | | | |  |
| **Selection**: scored a maximum of five stars | | | |  |
| **Comparability**: scored a maximum of two stars | | | |  |
| **outcome**: scored a maximum of three stars | | | |  |
| a score of ≥7 out of 10 were considered as achieving high quality | | | |  |
